# Wild Boars as Reservoir of Highly Virulent Clone of Hybrid Shiga Toxigenic and Enterotoxigenic *Escherichia coli* Responsible for Edema Disease, France

**DOI:** 10.3201/eid2802.211491

**Published:** 2022-02

**Authors:** Alexandre Perrat, Priscilla Branchu, Anouk Decors, Silvia Turci, Marie-Hélène Bayon-Auboyer, Geoffrey Petit, Vladimir Grosbois, Hubert Brugère, Frédéric Auvray, Eric Oswald

**Affiliations:** Institut de Recherche en Santé Digestive, Université de Toulouse, Institut National de la Santé et de la Recherche Médicale, Institut National de Recherche pour l’Agriculture, l’Alimentation et l’Environnement (INRAE), Ecole Nationale Vétérinaire de Toulouse, Université Paul Sabatier, Toulouse, France (A. Perrat, P. Branchu, H. Brugère, F. Auvray, E. Oswald);; Office Français de la Biodiversité, Orléans, France (A. Decors);; Labocea, Ploufragan, France (S. Turci, M.-H. Bayon-Auboyer);; Université Clermont Auvergne INRAE, VetAgro Sup, Unité Mixte de Recherche (UMR) Epidémiologie des Maladies Animales et Zoonotiques, Saint-Genès-Champanelle, France (G. Petit);; UMR Animal-Santé-Territoires-Risques-Ecosystèmes, Centre de Coopération Internationale en Recherche Agronomique pour le Développement, INRAE, Montpellier, France (G. Petit, V. Grosbois);; Centre Hospitalier Universitaire de Toulouse, Hôpital Purpan, Toulouse (E. Oswald)

**Keywords:** *Escherichia coli*, STEC, ETEC, EDEC, edema disease, Stx2e, O139:H1, F18, enterotoxin, wild boar, pigs, swine, *Sus scrofa*, *Sus domesticus*, France, bacteria, antimicrobial resistance

## Abstract

Edema disease is an often fatal enterotoxemia caused by specific strains of Shiga toxin–producing *Escherichia coli* (STEC) that affect primarily healthy, rapidly growing nursery pigs. Recently, outbreaks of edema disease have also emerged in France in wild boars. Analysis of STEC strains isolated from wild boars during 2013–2019 showed that they belonged to the serotype O139:H1 and were positive for both Stx2e and F18 fimbriae. However, in contrast to classical STEC O139:H1 strains circulating in pigs, they also possessed enterotoxin genes *sta1* and *stb*, typical of enterotoxigenic *E. coli*. In addition, the strains contained a unique accessory genome composition and did not harbor antimicrobial-resistance genes, in contrast to domestic pig isolates. These data thus reveal that the emergence of edema disease in wild boars was caused by atypical hybrid of STEC and enterotoxigenic *E. coli* O139:H1, which so far has been restricted to the wildlife environment.

First described in 1938, edema disease (ED) causes edema in various tissues of the domestic pig (*Sus scrofa domesticus*), characterized by neurologic disorders (ataxia, convulsions, incoordination, and lateral decubitus with paddling of limbs); swollen eyelids, forehead, and ears; a peculiar squeal; and sometimes sudden death (all usually without diarrhea or fever) (*1*). More commonly affecting pigs within 2 weeks after weaning, this disease can also occur in older pigs. The disease is the result of infection with a subset of Shiga toxin (Stx)–producing *Escherichia coli* (STEC), expressing plasmid-encoded F18 fimbriae and α-hemolysin (*hly*) and prophage-encoded Stx2e subtype (*2*,*3*). After F18-mediated STEC adhesion to the intestinal mucosa, Stx2e reaches the bloodstream and causes vascular damage in several target organs, commonly the brain and gastrointestinal tract (*4*,*5*). In Europe, ED-causing STEC strains mainly belong to the following serotypes (in order of importance): O139:K82:H1, O141:K85:H4, and O138:K81:NM (*1*,*6*,*7*). Outbreaks of ED caused by the O147 serogroup have also been reported in the United States (*8*).

Neonatal enteric colibacillosis and postweaning diarrhea (PWD) are other crucial factors contributing to death in nursery pigs in global swine production. These diseases are caused by enterotoxigenic *E*. *coli* (ETEC), which produce heat-stable toxins (STa, STb), heat-labile toxins (LT), or both. These toxins bind to specific receptors of the intestinal epithelial cells and cause secretion of water and electrolytes into the intestinal lumen. ETEC causing neonatal diarrhea typically produce F4, F5, F6, or F41 fimbriae, whereas those causing PWD produce either F4 or F18 fimbriae (*1*,*9*). The F4 receptors are expressed on porcine enterocytes irrespective of age, whereas F18 receptors are not fully expressed in pigs <3 weeks of age (*10*). Most PWD F4-positive ETEC are of the serogroup O149, whereas F18-positive ETEC belong to many serogroups, including O138, O139, O141, O147, and O157, because the F4 or F18 fimbriae gene cluster and enterotoxin genes are encoded on conjugative plasmids that result in their spread (*1*). Most of these strains are also hemolytic because the *hly* operon is frequently associated with fimbriae gene clusters on conjugative plasmids (*11*–*13*). Some F18-positive strains produce both enterotoxins and Stx2e (*1*,*11*) and thus belong to a hybrid STEC–ETEC pathotype.

In 2013, a total of 109 wild boars (*S. scrofa scrofa*) were suspected of being affected by ED in the southeast of France, thus corresponding to the first ED cases reported in wild boars living in natural environmental conditions (*14*). Other ED outbreaks occurred later in 2014 (51 cases), 2015 (26 cases), and 2016 (5 cases), as well as in 2019 (7 cases), in the same region. The boars were mainly 4–6 months old, corresponding to the weaning period in this species (*15*). Given the increase of the wild boar population in Europe in the last decades (*16*), which can lead to more frequent contact with domestic pigs and increasing risk for disease transmission (*17*), we characterized the strains responsible for the emergence of ED in wild boars. To this aim, we sequenced the whole genome of 28 wild boar STEC O139:H1 isolates from the different ED outbreaks and performed a genetic and genomic comparison with STEC O139:H1 and non-O139:H1 strains isolated from domestic pigs and other sources worldwide.

## Materials and Methods

### Bacterial Strains Analyzed 

We analyzed a collection of 28 STEC O139:H1 strains isolated in France from the intestinal content or lymph nodes, after necropsy, of wild boars with clinical signs and lesions consistent with ED, along with 16 STEC O139:H1 and 6 STEC O141:H4 strains isolated in France from pigs affected by ED (Appendix 1 Table 1). We also included in this study an additional 168 *E. coli* strains isolated from pigs or other sources, whose genome sequences were retrieved from the GenBank (*18*) and Enterobase (*19*) databases (Appendix 1 Table 2).

### Whole-Genome Sequencing

For short-read sequencing, we purified genomic DNA from 200 µL of lysogeny broth overnight cultures by using MagNA Pure 96 DNA and Viral NA Small volume Kit (Roche Molecular Systems Inc., https://www.roche.com). We then sequenced genomic DNA and generated 2 × 150 bp paired-end reads by using Illumina NextSeq500 (IntegraGen SA, https://integragen.com) with 80× coverage from libraries we obtained by enzymatic fragmentation by using a 5× whole-genome sequencing fragmentation mix kit (Enzymatics Inc., https://www.enzymatics.com).

We performed long-read sequencing for 3 strains (P13-6, P15-25, and W13-16) by using PacBio RSII system (GenoScreen SAS, https://www.genoscreen.fr) with 50× coverage. We extracted genomic DNA by using Gentra Puregene Yeast/Bact Kit (QIAGEN, https://www.qiagen.com) and prepared the libraries according to the protocol of SMRTbell Express Template Prep Kit 2.0 (PacBio, https://www.pacb.com) with selection of fragment size at 15–20 kb. We conducted an additional paired-end 2 × 100-bp Illumina MiSeq sequencing (GenoScreen SAS) with 50× coverage for these 3 strains by using genomic DNA extracted with Wizard Genomic DNA Purification Kit (Promega Corporation, https://www.promega.com) and libraries we prepared with a Nextera XT DNA Library Preparation Kit (Illumina, https://www.illumina.com).

### Genome Assembly and Phylogeny

We trimmed the raw sequencing reads by using TrimGalore 0.6.5 (https://www.bioinformatics.babraham.ac.uk/projects/trim_galore), then assembled them with Unicycler 0.4.8.0 (*20*), excluding contigs <100 bp, with a normal bridging mode. We combined long reads with short reads during assembly. We annotated each assembly by using Prokka 1.14.5 (*21*) with a similarity e-value cutoff of 1^−6^. We aligned the core genomes by using Roary 3.13.0 (*22*), with a minimum percentage identity of 95% for blastp (https://blast.ncbi.nlm.nih.gov/Blast.cgi?PAGE=Proteins), a minimum percentage of 99% isolates for genes included in the core genome, and Markov clustering inflation value of 1.5. For the O139-specific tree, we mapped the raw reads against the *E. coli* K-12 MG1655 reference strain by using Bowtie2 (*23*) and performed single-nucleotide polymorphism (SNP) calling by using BioNumerics 7.6.3 (bioMérieux, https://www.biomerieux.com), removing positions with >1 unreliable or ambiguous base and a minimum absolute coverage of 5. We generated the minimum-spanning tree with BioNumerics 7.6.3 and performed maximum-likelihood phylogenetic trees with IQ-TREE 1.5.5 (*24*). We built the tree of the entire collection by using a generalized time-reversible substitution model with an empirical base frequency and a FreeRate model of site heterogeneity (*25*,*26*) with 10 categories, whereas construction of the O139-specific tree applied a k3Pu substitution model (*27*), after we used ModelFinder (*28*) to identify the best-fitting model according to the Akaike information criterion. We compared the phylogenetic tree with the resistance factors and analyzed the phylogeography of the strains by using Microreact (*29*) and annotated the O139-specific tree by using FigTree 1.4.4 (https://github.com/rambaut/figtree). We produced chromosomal and plasmid maps by using BIG 0.95 (*30*). We submitted all sequence data generated in this study to the National Center for Biotechnology Information’s BioProject database (accession no. PRJNA741404).

### Composition of the Accessory Genome, Resistance Genes, and Virulence Genes

We detected virulence genes by using VirulenceFinder 2.0.3 (*31*) with a minimum percentage identity of 90% and resistance genes by using BioNumerics 7.6.3 with a minimum percentage identity of 85%, both with a minimum length of 60%. We subtyped F18 fimbriae by using amino acid sequence analysis of the major FedA subunit, including positions 122 and 123 (glycine and serine for F18ab, proline and alanine for F18ac) (*2*).

We analyzed the relationship between strains on the basis of accessory genome composition by using a t-distributed stochastic neighbor embedding (t-SNE) machine learning algorithm with Panini v1 (https://gitlab.com/cgps/panini/bhtsne), with a gradient accuracy (theta) of 0.5 and an auto perplexity (p). Using the table of genes present or absent in the strains of the entire collection outputted from the Roary pipeline, we conducted pan-GWAS analysis to measure the statistical significance of the association of certain genes with the clade of wild boar strains by using Scoary 1.6.16 (https://github.com/AdmiralenOla/Scoary). We retained the annotated genes with a p value <2.21 × 10^−12^ by Fisher exact test. 

### Stx2e Phages, Plasmids, and Pairwise Comparison

We detected phages by using Phaster (*32*). We extracted the sequences corresponding to the Stx2e phage and circular contigs (plasmids) from hybrid assemblies. We retrieved the closest similar plasmid sequence available online from the National Center for Biotechnology Information nucleotide collection (nr/nt) database (accessed April 1, 2020). We then compared Stx2e phage and plasmid sequences by using blastn 2.9.0 (https://blast.ncbi.nlm.nih.gov/Blast.cgi?PROGRAM=blastn&PAGE_TYPE=BlastSearch&LINK_LOC=blasthome) with default parameters, along with GenBank annotated sequences, to create pairwise comparison in EasyFigure 2.2.3 (*33*).

### Antimicrobial-Susceptibility Testing

We determined antimicrobial drug susceptibility profiles of the 3 PacBio-sequenced strains (P13-6, P15-25, and W13-16) and 3 other *E. coli* strains (W14-3, W15-17, and W19-4) by using the Vitek 2 system (bioMérieux). We interpreted MIC results for ampicillin, ticarcillin, piperacillin/tazobactam, cefalotin, cefoxitin, cefotaxime, ceftazidime, ertapenem, imipenem, amikacin, gentamicin, tobramycin, nalidixic acid, ciprofloxacin, ofloxacin, nitrofurantoin, trimethoprim/sulfamethoxazole, erythromycin, tetracycline, and chloramphenicol according to the 2020 criteria of the European Committee on Antimicrobial Susceptibility Testing (https://www.eucast.org).

## Results

### Core Genome–Based Phylogenetic Analysis

**Figure 1:**
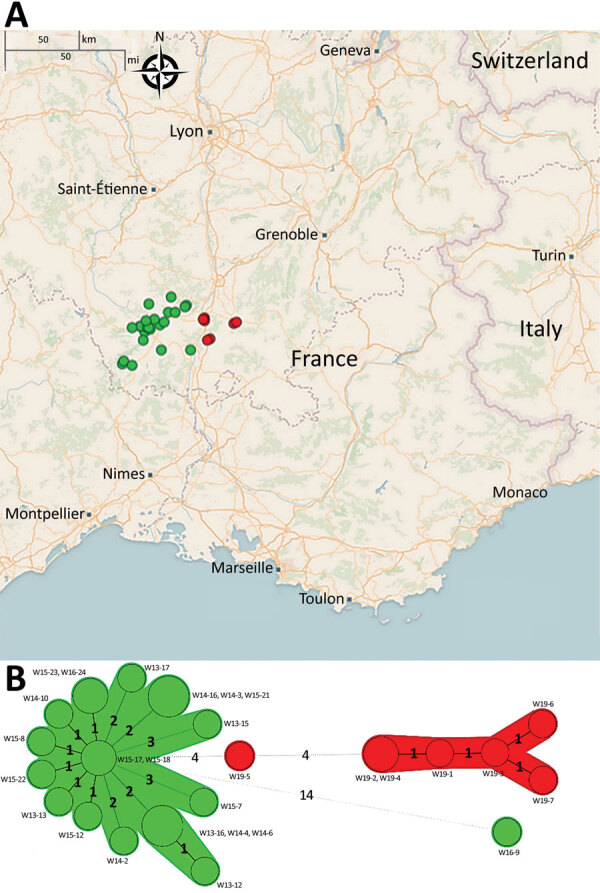
Geographic location of 28 wild boar *Escherichia coli* O139:H1 strains in France (A) and phylogeny represented as a minimum spanning tree (B) using BioNumerics 7.6.3 (bioMérieux, https://www.biomerieux.com). Sizes of the discs represent number of isolates. Colors of the discs represent year of isolation (green, 2013–2016; red, 2019). Numbers of differing single-nucleotide polymorphisms (SNPs) are indicated on connecting lines between the nodes.

**Figure 2:**
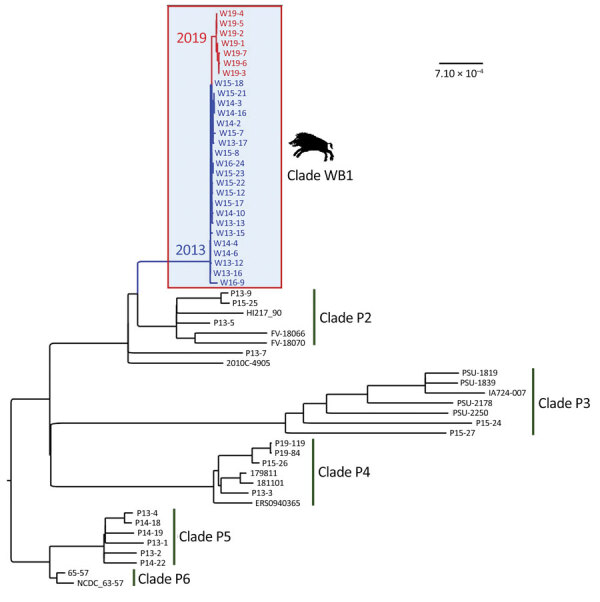
Core genome maximum-likelihood phylogenetic tree of 63 *Escherichia coli* O139:H1 (ST1) isolates from edema disease cases, including 28 from wild boars in France and 35 from domestic pigs of worldwide origin, including France. The clade of wild boar strains (WB1) is boxed, and the strains from this clade are colored according to the year of isolation (blue, 2013–2016; red, 2019). The clades of pig strains are numbered from P2 to P6. Scale bar indicates the number of substitutions per site.

We performed short-read whole-genome sequence analysis of 28 STEC O139:H1 strains isolated from wild boars that had clinical signs and lesions consistent with ED during multiple outbreaks that occurred in the southeast of France: in the Ardèche Department in 2013 (n = 5), 2014 (n = 6), 2015 (n = 8), and 2016 (n = 2) and in the Drôme Department in 2019 (n = 7) (Appendix 1 Table 1). These strains were phylogenetically close based on SNP analysis (Figure 1), most of them showing <10 SNP differences considered as the threshold to determine strain relatedness (*34*). The most genetically distant isolates corresponded to an Ardèche isolate from 2016 and 6 Drôme isolates from 2019 (Figure 1), suggesting an increase of genetic variability over time, space, or both. We enlarged the phylogenetic analysis to include 35 *E. coli* O139:H1 isolates from domestic pigs of worldwide origin, including France. The core genome–based maximum-likelihood tree showed that the 28 wild boars STEC O139:H1 strains clustered into a distinct clade (named WB1) (Figure 2). This first level of analysis indicated that the STEC strains isolated from the different ED outbreaks in wild boars corresponded to a single *E. coli* clone of serotype O139:H1.

### Genomic Features of Wild Boar *E. coli* O139:H1 Compared to Porcine *E. coli* O139:H1 and O141:H4

We used long-read sequencing for strain W13-16 to provide a closed genome for a representative strain of STEC O139:H1 isolated from wild boars (chromosome and plasmid maps in Appendix 2 Figure). We compared that genome with the long-read sequenced genomes obtained for pig ED STEC strains P15-25 and P13-6, which belonged to the 2 serotypes most commonly reported in ED cases in France (O139:H1 for P15-25, O141:H4 for P13-6) (*6*). Strain W13-16 contained 2 plasmids of 54.7 and 83.4 kb, whereas P15-25 contained 1 plasmid of 77.5 kb and P13-6 contained 9 plasmids with sizes ranging from 3.1 to 226.4 kb (Table; Appendix 2 Figure).

**Table:** Genomic characteristics of chromosomes and plasmids of wild boar *Escherichia coli* strain W13–16 and pig *E. coli* strains P15-25 and P13-6, France*

Strain and support	Length, bp	Typing	Resistance inventory	Virulence inventory
W13-16				
Chromosome	5,091,917	O139:H1 ST1	*mdf(A)*	*chuA*, *ehaG*, *eilA*, *kps*, LPF, *ompT*, *rhsA stx*, *terC*, T6SS, *vgrG1*
pW1316-1	83,443	IncFII [F10:A-:B-]		*aidA*, *sta1*, *stb*, *sepA*
pW1316-2	54,694	IncFII/IncX1 [F14:A-:B-]		*aidA-I*, α-*hly,* F18
P15-25				
Chromosome	5,029,591	O139:H1 ST1	*mdf(A)*	*chuA*, *eilA*, *kps*, LPF, *ompT*, *rhsA*, *stx*, *terC*, T6SS, *vgrG1*
p1525-1	77,484	IncFII/IncX1 [F14:A-:B-]		*aidA*, *aidA-I,* α-*hly,* F18
P13-6				
Chromosome	4,963,420	O141:H4 ST10	*mdf(A)*	*bcs*, ETT2, *iss*, *ompT*, *stx*, *terC*
pP136-1	226,437	IncHI2 DLST:ST4	*mph(B)*, *tetR*	*terC*
pP136-2	103,673	IncI1-I(Alpha) ST26/CC2	*aadA1*, *aadA2*, *cmlA1*, *mef(B*), *sul3*	*cib*
pP136-3	86,378	IncFII/IncX1 [F14:A-:B-]		*aidA-I*, α-*hly,* F18
pP136-4	82,875	IncFII [F108:A-:B-]		F4
pP136-5	82,610	IncFII [F10:A-:B-]		*aidA*, *sta1*, *stb*, *sepA*
pP136-6	74,646	p0111		
pP136-7	48,077			
pP136-8	5,125			
pP136-9	3,126			

*Serotype and ST are indicated for the chromosomes, whereas incompatibility group, FAB [FII, FIA, FIB] formulas, and ST or CC are indicated for plasmids. CC, clonal complex; ST, sequence type.

**Figure 3:**
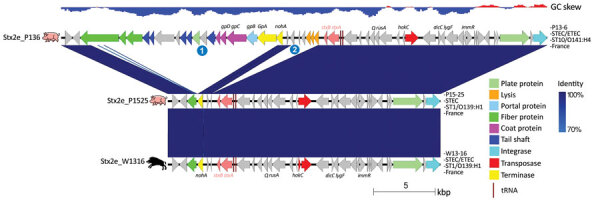
Comparison of the Stx2e prophages of wild boar *Escherichia coli* O139:H1 strain W13-16 and pig *E. coli* O139:H1 P15-25 and O141:H4 P13-6 strains from France. The genes are represented with arrows color coded by function. The 2 regions present in prophage Stx2e_P136 but absent in the 2 other prophages are indicated by numbers 1 and 2. The areas between the genetic maps are shaded in blue, with a color intensity depending on the percentage of identity between each region compared. Strain name, pathotype, sequence type, serotype, and country of isolation are indicated at the right of each map. The GC skew (negative, blue; positive, red) is indicated at the top. ETEC, enterotoxigenic *Escherichia coli;* ST, sequence type; STEC, Shiga toxin–producing *Escherichia coli*.

The chromosome of the STEC W13-16 strain carried an Stx2e prophage (Table; Appendix 2 Figure) whose sequence was highly similar to those of the 2 porcine STEC O139:H1 and O141:H4 strains, except for 2 phage regions that were deleted in both STEC O139:H1 isolates, in contrast to STEC O141:H4 (Figure 3). These 2 regions contained several late genes involved in the phage lytic cycle and more precisely in the assembly of the head, collar, fibers, and tail (region 1) and lysis (region 2) (Figure 3). Such deletions thus probably result in deficiency of STEC O139 for the production of Stx2e phage particles, as observed previously for many other *stx2e*-positive *E. coli* strains whose Stx2e phages were shown to lack >1 genes and to be not inducible (*35*,*36*).

**Figure 4:**
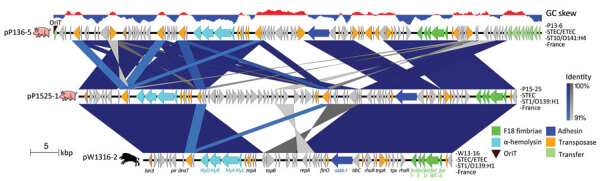
Comparison of plasmids carrying the F18 fimbriae gene cluster of the wild boar *Escherichia coli* O139:H1 strain W13-16 and pig *E. coli* O139:H1 P15-25 and O141:H4 P13-6 strains. The genes are represented with arrows color coded by function. The areas between the genetic maps are shaded in blue or gray for regions oriented in the same or opposite direction, respectively, with a color intensity depending on the percentage of similarity between each region compared. Strain name, pathotype, sequence type, serotype, and country of isolation are indicated at the right of each map. The GC skew (negative, blue; positive, red) is indicated at the top. ETEC, enterotoxigenic *Escherichia coli;* OriT, transfer origin; ST, sequence type; STEC, Shiga toxin–producing *Escherichia coli*.

We identified a plasmid, pW1316-2, classically found in STEC O139:H1 and encoding F18 fimbriae, Hly, and adhesin AidA-1, in strain W13-16 and assigned it to the incompatibility (Inc) group IncFII/IncX1 (Table). Previous reports showed that F18-positive plasmids from porcine STEC or ETEC strains possessed a replicon of the RepFIc/RepFIIa family (*37*) and that IncX1, IncI1, and IncFII plasmids are frequently encountered within F18-positive ETEC (*38*). F18-positive plasmids are also known to contain *hly* and *aidA* genes (*13*,*39*). Plasmid pW1316-2 possessed the F18ab antigenic variant, as previously observed for porcine ED STEC O139, in contrast to PWD ETEC from other serogroups (including O141), which produce F18ac (*40*). This plasmid displayed similarity with the IncFII/IncX1 plasmids pP1525 (F18ab-positive) from the pig STEC O139:H1 strain and pP136-5 (F18ac-positive) from the pig STEC O141:H4 strain (Table; Figure 4). However, these 2 plasmids were larger than pW1316-2 and contained additional regions with open reading frames of unknown function (Figure 4). We identified no transfer region in the 3 F18-positive plasmids pW1316-2, pP1525, and pP136-5 (Figure 4), suggesting that they are transfer-deficient. Only 2 closed F18-positive plasmid sequences have been described in the literature, both from non-O139 strains: an IncFIIA plasmid (pUMNF18_87, 87 kb) from a diarrheic pig STEC/ETEC O147 strain, carrying F18ac, *hly,* *aidA-1* genes and remnants of an F transfer region (*12*); and an IncFII/IncX1 plasmid (p15ODTXV, 119 kb) from a diarrheic pig STEC/ETEC O141:H4 strain, carrying F18ac, *hly,* and *sta*/*stb* genes and a conjugation transfer region (*11*).

**Figure 5:**
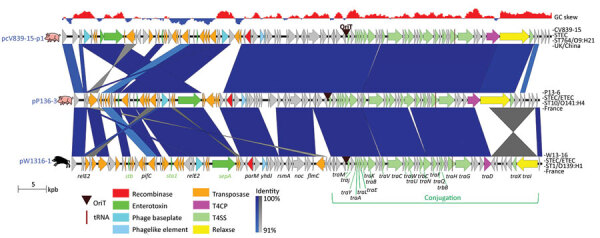
Comparison of plasmids carrying the enterotoxin and *sepA* virulence genes of the wild boar *Escherichia coli* O139:H1 strain W13-16 and pig *E. coli* O141:H4 P13-6 and O9:H21 CV839-15 strains from France. The genes are represented with arrows color coded by function. The areas between the genetic maps are shaded in blue or gray for regions oriented in the same or opposite direction, respectively, with a color intensity depending on the percentage of similarity between each region compared. Strain name, pathotype, sequence type, serotype, and country of isolation are indicated at the right of each map. The GC skew (negative, blue; positive, red) is indicated at the top. ETEC, enterotoxigenic *Escherichia coli;* ST, sequence type; STEC, Shiga toxin–producing *Escherichia coli*.

Surprisingly, the second plasmid of W13-16 (pW1316-1) (Table) was not classically found in STEC strains of serotype O139:H1. It belonged to the IncFII group and carried *sta1* and *stb* enterotoxin genes as well as the serine protease autotransporter SepA toxin gene and a second *aidA* gene (Table; Figure 5). The *sta1*/*stb* and *sepA* genes were bordered by many transposase genes and insertion sequence (IS) elements (Figure 5). Plasmid-encoded enterotoxins are a typical feature of porcine PWD ETEC strains, and enterotoxin genes surrounded by IS were also reported elsewhere (*12*,*37*,*41*), suggesting that IS may favor the acquisition of virulence genes. We did not find such a plasmid in the pig STEC O139:H1 strain, in contrast to the pig STEC O141:H4 strain, which carried a similar IncFII plasmid, pP136–3 (Table; Figure 5). A BLAST search (https://blast.ncbi.nlm.nih.gov/Blast.cgi) led to the identification of another similar plasmid (pCV839-15-p1) in a typical diarrheic pig ETEC strain of serotype O9:H21 (GenBank accession no. SAMN0804056) (Figure 5). Sequence comparison of plasmids pW1316-1, pP136-3, and pCV839-15-p1 showed that a highly conserved conjugation region was located downstream of the transfer origin. However, the region spanning the relaxase gene up to the type 4 coupling protein gene was reversed in pW1316-1 (Figure 5), resulting in truncation of the N-terminal part of the relaxase gene and the C-terminal part of the type 4 coupling protein gene, and presumably in conjugation deficiency.

On the basis of this genomic analysis, the wild boar W13-16 isolate should thus be considered as an atypical hybrid STEC–ETEC of the serotype O139:H1. We identified the *sta1*, *stb*, and *sepA* genes in all the O139:H1 isolates from clade WB1, except for 1 strain (W15-12), which was lacking these genes (Appendix 1 Table 3), presumably because of the loss of the plasmid carrying these virulence genes. In most O139:H1 isolates from pigs or other sources, the *sta*, *stb*, and *sepA* genes were lacking (Appendix 1 Table 3), indicating that the plasmid pW1316-1 conferring the hybrid STEC–ETEC status to the strains from clade WB1 is absent from O139:H1 strains of non–wild boar origin. By contrast, we frequently encountered the hybrid STEC–ETEC status in other *E. coli* serotypes, such as O138:H14, O141:H4, and O147:H4 (Appendix 1 Table 3).

### Comparing Global Composition of Entire Accessory Genome among *E. coli* O139:H1, O141:H4, O147:H4, and O138:H14 Strains

**Figure 6:**
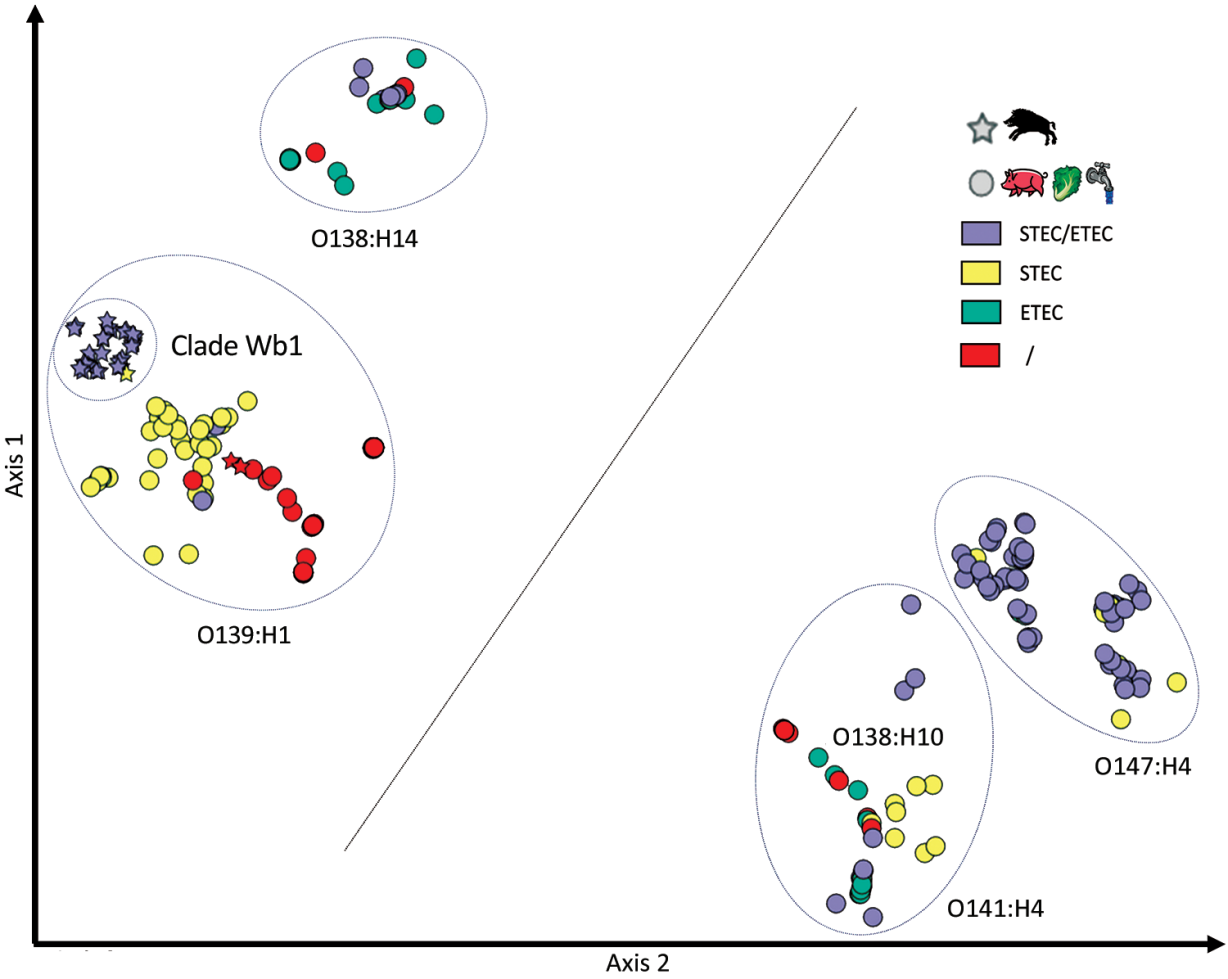
Comparison of the accessory genome composition of wild boar *Escherichia coli* O139:H1 strains in France with that of *E. coli* O139:H1, O141:H4, O147:H4, and O138:H14 of worldwide origin. Each sign represents a strain depending on its origin (star, wild boar; circle, other hosts). The distance between the signs in a 2-dimensional space increases with the decrease in orthologous genes in common between strains represented. The signs are color coded depending on the predicted pathotype. The 28 wild boar O139:H1 strains are represented by gray stars except for 1 strain lacking *sta1*/*stb* genes, represented by a yellow star. Two additional wild boar O139:H1 strains were included in this analysis and are represented by red stars because they lacked the *stx2e* and *sta1*/*stb* genes (Appendix 1 Table 3); they did not belong to clade WB1 (data not shown). ETEC, enterotoxigenic *Escherichia coli;* STEC, Shiga toxin–producing *Escherichia coli*; /, neither STEC nor ETEC nor hybrid STEC–ETEC.

We analyzed the presence of the *E. coli* virulence genes found in the STEC O139:H1 W13-16 strain in the other wild boar O139:H1 strains as well as in 190 additional *E. coli* strains originating in France or worldwide (Appendix 1 Tables 1, 2). These belonged to O139:H1, O141:H4, O147:H4, and O138:H14 serotypes and to various pathotypes (i.e., STEC, ETEC, hybrid STEC–ETEC, or none of these) depending on the presence or absence of *stx* and *sta1*/*stb* virulence genes (Appendix 1 Table 3). By analyzing the global composition of the accessory genome, we found that all these strains clustered into 4 main groups, consistent with the 4 major serotypes (Figure 6).

Among the accessory genome, certain genes were significantly associated, although not exclusively, with the strains of clade WB1, such as *rhsA*, which encodes an effector of the type 6 secretion system (T6SS) (*42*) and the gene coding for the trimeric autotransporter adhesin EhaG (*43*) (Appendix 1 Table 3). As mentioned previously, the SepA encoding gene was predominant in strains of clade WB1 and quite rare in the other strains of *E. coli* responsible for ED. SepA, originally described in *Shigella flexneri* 2a and enteroaggregative *E. coli*, has been identified only in F4-positive ETEC strains isolated from pigs (*38*,*44*), where it was shown to be also encoded on a large (85 kb) plasmid (*45*). SepA, a serine protease autotransporter of the *Enterobacteriaceae*, could degrade intestinal mucin (*46*).

### Antimicrobial-Resistance Genotypes and Phenotypes

**Figure 7:**
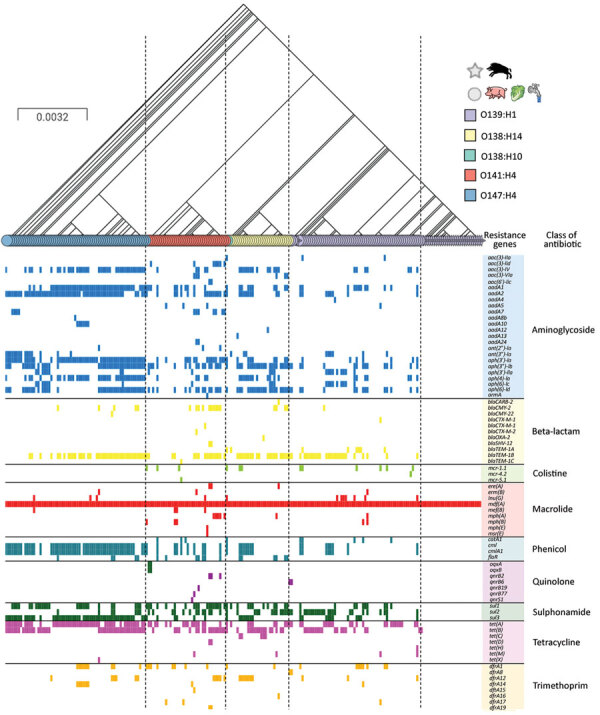
Comparison of antimicrobial-resistance genes with the phylogeny of wild boar *Escherichia coli* O139:H1 strains in France with those of *E. coli* O139:H1, O141:H4, O147:H4, and O138:H14 of worldwide origin. The tree is based on the phylogeny of the strains according to their core genome. The shapes of the leaves in the tree correspond to the origin of the strains (star, wild boars; circle, other hosts), and the colors of the leaves represent their serotype. Antimicrobial-resistance genes are grouped into different categories whose names are indicated at the top, with a color code. Scale bar indicates number of substitutions per site.

The O139:H1 strains of clade WB1 did not carry any gene involved in resistance to classical antibiotics except that of the efflux pump *mdf*(*A*), which can confer resistance to macrolides and is found in most *E. coli* strains (Figure 7). By contrast, the O139:H1 strains from porcine origin carried a high amount of antimicrobial-resistance genes, which was also the case for porcine O138:H14, O141:H4, and O147:H4 strains. Except for a minority of isolates, in most pig strains we identified genes conferring resistance to various classes of antibiotics, including aminoglycosides, β-lactam, colistin, macrolide, phenicol, quinolone, sulphonamide, tetracycline, and trimethoprim (Figure 7).

The antimicrobial-susceptibility testing of 4 wild boar STEC O139:H1 isolates (W13-16, W14-3, W15-17, and W19-4) recovered from different years confirmed the results of the in silico analysis because they were sensitive to all antibiotics tested except for erythromycin. We also tested the 2 pig O139:H1 (P15-25) and O141:H4 (P13-6) strains whose closed genomes we obtained. P15-25 was sensitive to all antibiotics tested except for erythromycin, consistent with the presence of the chromosomal *mdf*(*A*) gene and absence of other antimicrobial-resistance gene on its single plasmid, pP1525. By contrast, P13-6 was resistant to erythromycin, tetracycline, and chloramphenicol, consistent with the presence of plasmid genes *mef(B)* and *tetRACD* (pP136-1) and *cmlA1* (pP136-2), as mentioned previously in our description of plasmids.

## Discussion

We show that the STEC O139:H1 strains that caused ED in wild boars in France belong to a specific clade (WB1) of *E. coli* O139:H1 strains that is similar, by virtue of its core genome and F18-encoding plasmid, to clades of pathogenic *E. coli* O139:H1 from domestic pigs but is distinguished from them by the presence of an enterotoxin-encoding plasmid usually found in other *E. coli* serotypes typical of PWD. Indeed, our study rarely found enterotoxin genes in STEC O139, in contrast to non-O139 STEC or ETEC serogroups such as O138 or O141, as reported previously (*10*,*40*). These findings may invite speculation that this enterotoxin-encoding plasmid was acquired by an ancestor of clade WB1 strains from a non-O139 strain, through horizontal gene transfer. In support of this hypothesis, this plasmid displayed similarities with those found in pig strains of serotypes O9:H21 and O141:H4.

Except for the efflux pump *mdf(A)*, the strains from clade WB1 lacked antimicrobial-resistance genes, which contrasted drastically with the situation in pig strains overwhelmingly carrying multiple resistance cassettes (*9*). This finding could indicate that the clade WB1 was under low pressure to select antimicrobial-resistance genes during its recent evolutionary history. This pathogenic clade appears to be endemic to the territory of France and restricted to a wild boar population. From the analysis of the *FUT1* gene regulating the expression of the F18 receptor, the wild boar populations in France were found genetically susceptible to ED (*15*). Production of various virulence factors, including F18 adhesin, Stx2e, and enterotoxins, may be cited to explain the emergence of ED in wild boars because such a combination may confer increased virulence to the strains. In addition to the hybrid STEC–ETEC status, the possession of a specific accessory genome could also be responsible for the adaptation of this clade to wild boar hosts and their environment.

In conclusion, our results argue in favor of a new clade of ED-causing STEC that originated from wildlife and did not result from contacts between wild boars and domestic pigs. ED is thus not restricted to pigs, as usually described, and wild boars are also susceptible hosts. Because the wild boar population is growing and outdoor pig farming is rapidly developing in Europe because of animal welfare considerations, contacts between wild boars and pigs could enable the spread of infectious diseases, if appropriate biosecurity measures are not implemented (*47*). Surveillance of this highly pathogenic clade in the wild boar population and in livestock animals is therefore of the highest importance and is needed to study its spread in the wildlife reservoir and potential transmission to domestic pigs.

Appendix 1 Additional information about *Escherichia coli* strains selected for genetic and genomic comparison.

Appendix 2 Additional information about chromosomes and plasmids of *Escherichia coli* strains analyzed in this study.
