# Supplementary material for: Wild Boars as Reservoir of Highly Virulent Clone of Hybrid Shiga Toxigenic and Enterotoxigenic Escherichia coli Responsible for Edema Disease, France
Source: Emerg Infect Dis. 2022 Feb;28(2):382–93. doi: 10.3201/eid2802.211491 (PMC8798679; doi:10.3201/eid2802.211491)
Supplement: Appendix 2 — Additional information about chromosomes and plasmids of Escherichia coli strains analyzed in this study. [file 21-1491-Techapp-s2.pdf]

Article DOI: https://doi.org/10.3201/eid2802.211491

Wild Boars as Reservoir of Highly Virulent Clone of Hybrid Shiga Toxigenic and Enterotoxigenic *Escherichia coli* Responsible for Edema Disease, France

Appendix 2

Appendix 2 Figure (following pages). Circular chromosomal (A and B) and plasmid (C–F) maps of wild boar *E. coli* O139:H1 W13-16, pig *E. coli* O139:H1 P15-25 and pig *E. coli* O141:H4 P13-6. Color key codes for virulence genes, housekeeping genes, genes cluster and G+C content are indicated in the insets. For each map, the circles show, from the inside out, the nucleotide sequence positions (in kb), the GC skew (negative, green; positive, purple) and the location of genes and gene clusters of interest. Besides the two plasmids encoding F18 fimbriae and enterotoxins, seven additional plasmids (Table in main text, https://wwwnc.cdc.gov/EID/article/28/2/21-1491-T1.htm) were identified in the pig *E. coli* O141:H4 strain. These included the two large plasmids pP136-1 (IncHI2) and pP136-2 (IncI1-I Alpha), carrying resistance genes to antibiotics such as *mph(B)* (macrolides) in the case of pP136-1, and *mef(B)* (macrolides), *cmlA1* (chloramphenicol), *sul3* (sulfamethoxazole) and *aadA1* and *aadA2b* (streptomycin-spectinomycin) in the case of pP136-2 (Table in main text).

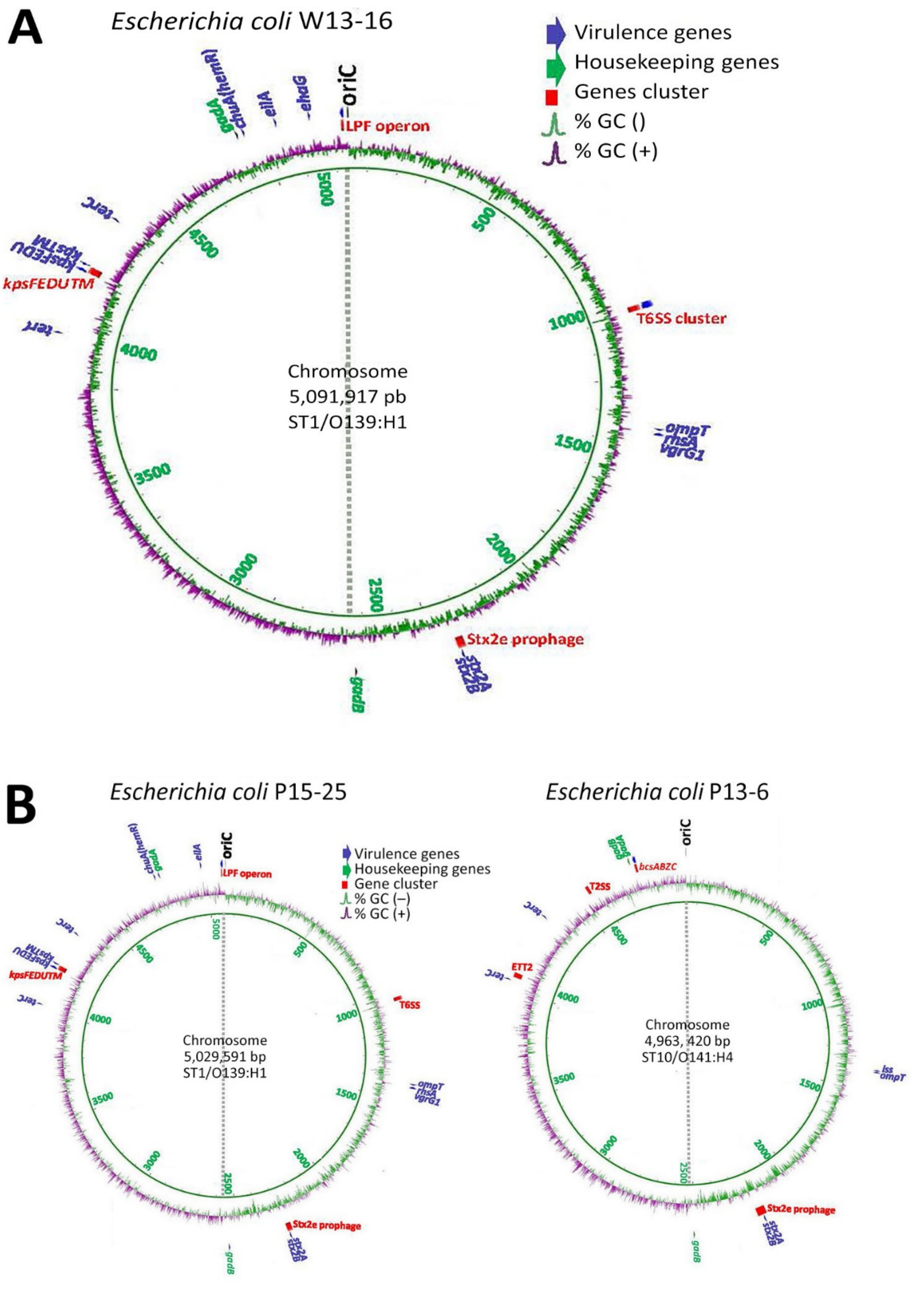
A
Escherichia coli W13-16
Virulence genes
Housekeeping genes
Genes cluster
% GC ()
% GC (+)
oriC
LPF operon
gadA
chuA(hemR)
eilA
ehaG
terC
kpsFEDU
kpsTM
kpsFEDUTM
T6SS cluster
ompT
rhsA
vgrG1
Stx2e prophage
stx2A
stx2B
gadB
Chromosome
5,091,917 pb
ST1/O139:H1
B
Escherichia coli P15-25
Escherichia coli P13-6
Virulence genes
Housekeeping genes
Gene cluster
% GC (–)
% GC (+)
Chromosome
5,029,591 bp
ST1/O139:H1
T6SS
bcsABZC
T2SS
ETT2
Chromosome
4,963, 420 bp
ST10/O141:H4
lss
ompT

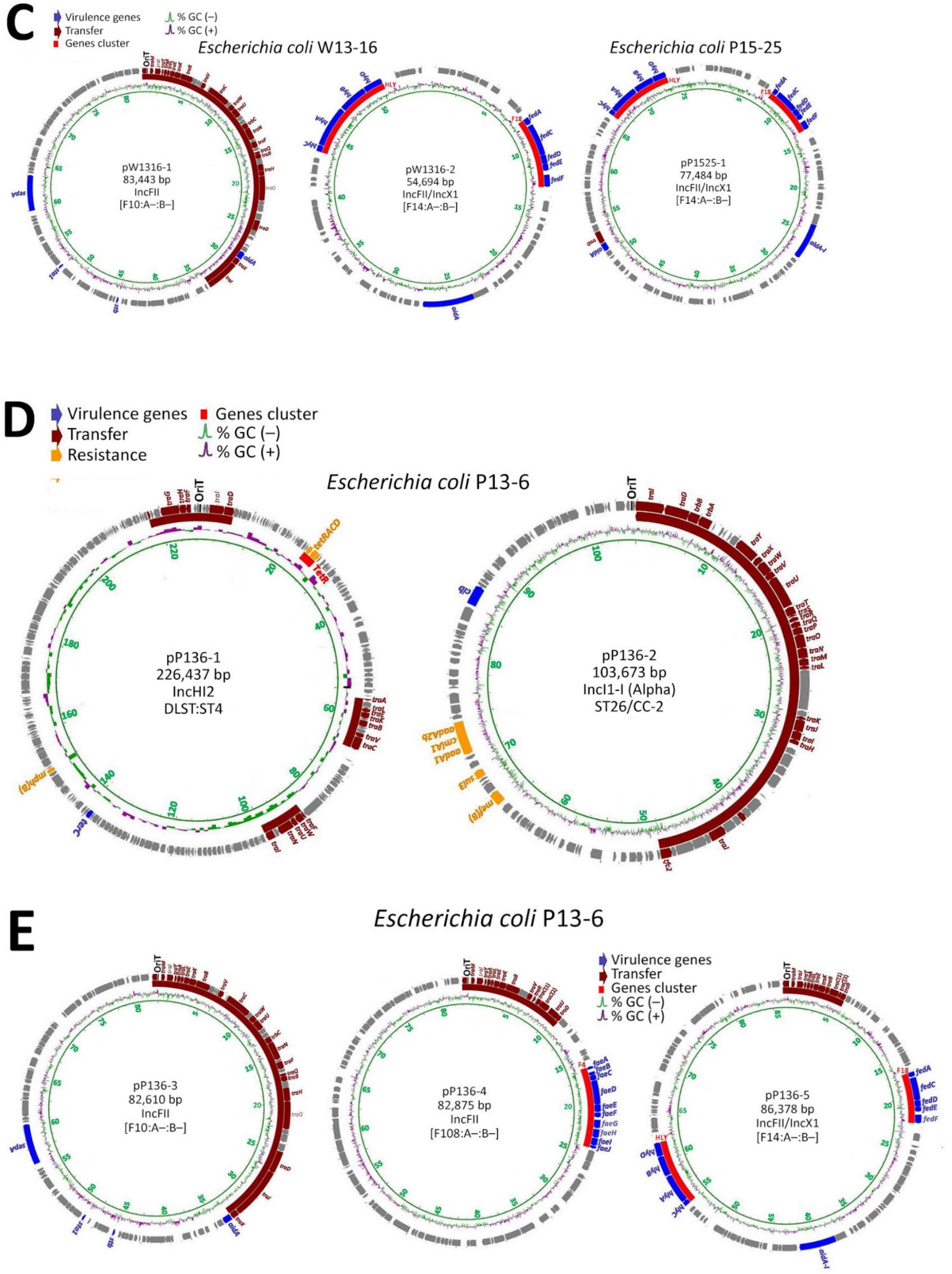
C
Virulence genes
Transfer
Genes cluster
% GC (–)
% GC (+)
Escherichia coli W13-16
Escherichia coli P15-25
pW1316-1
83,443 bp
IncFII
[F10:A–:B–]
pW1316-2
54,694 bp
IncFII/IncX1
[F14:A–:B–]
pP1525-1
77,484 bp
IncFII/IncX1
[F14:A–:B–]
D
Virulence genes
Transfer
Resistance
Genes cluster
% GC (–)
% GC (+)
Escherichia coli P13-6
pP136-1
226,437 bp
IncHI2
DLST:ST4
pP136-2
103,673 bp
IncI1-I (Alpha)
ST26/CC-2
E
Escherichia coli P13-6
Virulence genes
Transfer
Genes cluster
% GC (–)
% GC (+)
pP136-3
82,610 bp
IncFII
[F10:A–:B–]
pP136-4
82,875 bp
IncFII
[F108:A–:B–]
pP136-5
86,378 bp
IncFII/IncX1
[F14:A–:B–]

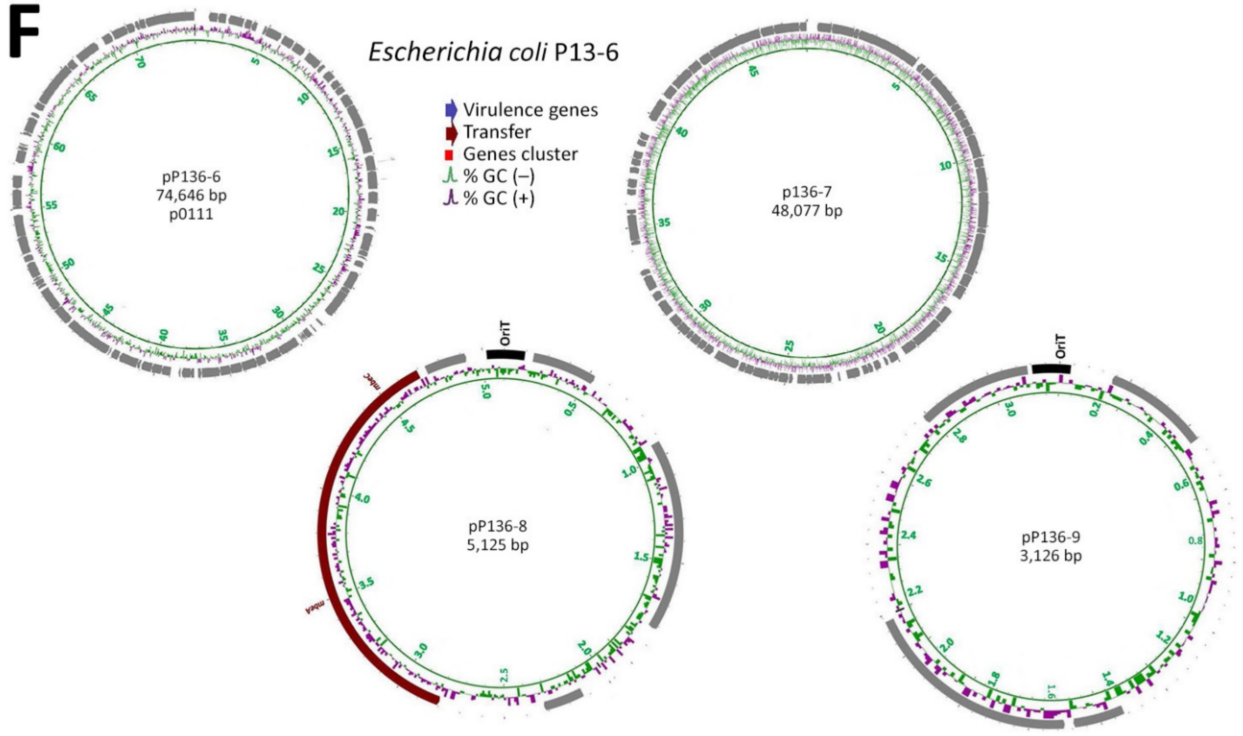
F
Escherichia coli P13-6
Virulence genes
Transfer
Genes cluster
% GC (–)
% GC (+)
pP136-6
74,646 bp
p0111
p136-7
48,077 bp
pP136-8
5,125 bp
OriT
pP136-9
3,126 bp
OriT
